# Supplementary material for: Local Volume Concentration, Packing Domains and Scaling Properties of Chromatin
Source: ArXiv. 2024 Mar 5:arXiv:2310.02257v2. Preprint. [Version 2] (PMC10942481)
Supplement: Supplementary file 1 [file NIHPP2310.02257V2-supplement-1.pdf]

Appendix 1

Supplementary Videos

Three supplemental videos are included:

- **Video V1**: SR-EV configuration represented as beads and sticks, wrapped in a mesh envelope that separates the dense regions from the nearly empty regions of the configuration. The beads have a diameter to 25% of their actual size to allow for more visibility. This configuration is the same as the one displayed on Figure 2-E and 2-G. The configuration rotates about the Z-axis to give a good understanding of its corrugated character.
- **Video V2**: Representation of the same system of Video V1, but in this case it contains only the wrapping mesh that resembles the interface between two disordered bi-continuous phases.
- **Video V3**: Stack of images from a conformation obtained with $\alpha = 1.20$ and $\phi = 0.12$. The planes are separated by 5 nm, and in plane resolution is 2 nm × 2 nm. The image shows the variability of 2D representation in a 100 nm slab.

Appendix 2

Supplementary Figures

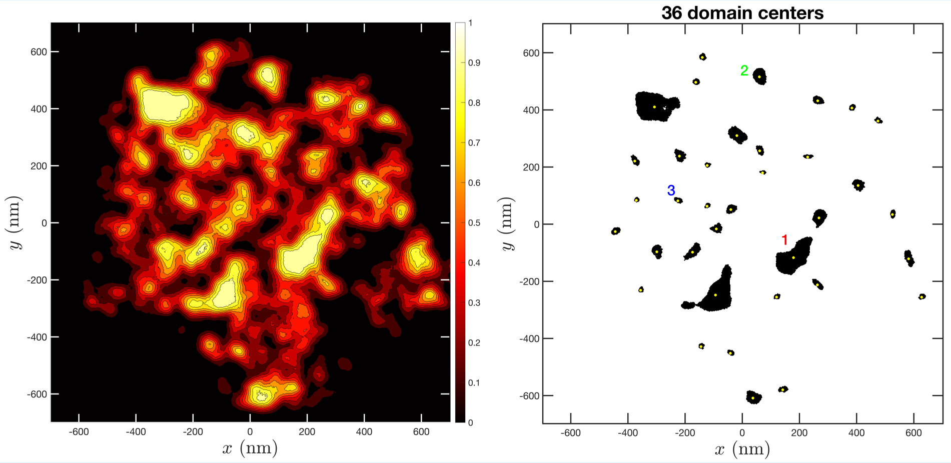

Appendix 2—figure 1. Example of domain and domain's center determination from SR-EV slabs. The left image shows the collapse SR-EV density from a 100 nm slab. The right image shows the identified domains cores in black and their geometric centers in yellow. Three different domains are identified with the numbers.

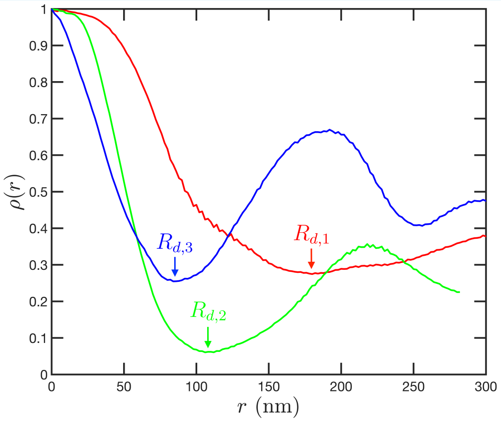

Appendix 2—figure 2. Example of the determination of the density profiles of domains and their effective radii. The three cases correspond to the large, medium and small domains denoted by 1, 2 and 3 in Figure S1. The profiles are calculated from the domain center using the coordinates from the configurations and assuming cylindrical symmetry. The radius of a domain corresponds to the first minimum in the density profile.

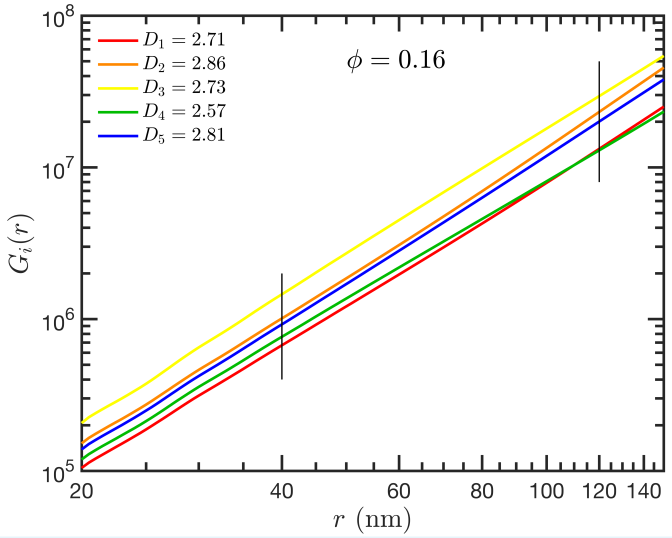

Appendix 2—figure 3. Example of cumulative distribution functions, $G_i(r)$, for five different SR-EV configurations. Each $G_i(r)$ is fitted with a power law between 40 and 120 nm to determine the packing coefficient D_i corresponding to that configuration.

Appendix 3

Supplementary Algorithms

Algorithm for the generation of a SRRW in free space

Here we describe a recursive Monte Carlo algorithm to generate a SRRW parameterized by folding parameter $\alpha > 1$ and local cutoff $U_{\rm max}$, which represents the maximum bond length. Within the algorithms to be described, the length unit is the minimum bond length $U_{\rm min} = 10$ nm, so that all length are dimensionless, and taken relative to the minimum bond length $U_{\rm min}$. The conformation of the SRRW, emanating from the origin, is defined by N bond vectors $\mathbf{U}_1$, $\mathbf{U}_2$, ..., $\mathbf{U}_N$. In the following, the symbol ξ stands for an independent random number drawn with equal probability from the interval $[0,1]$, and has to be recreated whenever it occurs below.

(A1) Define $\beta \equiv 1 - U_{\rm max}^{-(1+\alpha)}$.
(A2) Generate a set of $2N$ bond vectors $\mathbf{B}_n$ with $n = 1,2,..,2N$ for eventual later use. Each $\mathbf{B}_n$ is given by $\mathbf{B}_n = \ell \mathbf{u}$, where $\mathbf{u}$ is a random unit vector and $\ell = (1-\beta\xi)^{-1/(1+\alpha)}$ its bond length. A random unit vector we create via $\mathbf{u} = (\sqrt{1-z^2}\cos\phi, \sqrt{1-z^2}\sin\phi, z)$, where $\phi = 2\pi\xi$ and $z = 2\xi - 1$. The generation of the set $\{\mathbf{B}\}$ hence requires $6N$ random numbers ξ and if not otherwise mentioned, the $\{\mathbf{B}\}$ will remain unchanged during the course of the algorithm.
(A3) Initialize $n = N$, set $\mathbf{U}_1 = \mathbf{B}_n$.
(A4) Increase n by one, set $\mathbf{U}_2 = \mathbf{B}_n$, and initialize step $s = 2$.
(A5) Call a recursive routine that takes the existing sets $\{\mathbf{B}\}$, $\{\mathbf{U}\}$, n, and s as arguments, and returns new sets $\{\mathbf{B}\}$, $\{\mathbf{U}\}$, and n. This routine does the following:

    (i) If $s = N$, just return from the routine.
    (ii) Calculate return probability $P_R = |\mathbf{U}_s|^{-\alpha}/\alpha$.
    (iii) If $\xi < P_R$, then $\mathbf{U}_{s+1} = -\mathbf{B}_n$ and n is decreased by one. Otherwise, n is increased by one, the single $\mathbf{B}_n$ is re-created using the above procedure (A2), and $\mathbf{U}_{s+1} = \mathbf{B}_n$.
    (iv) Routine calls itself with identical arguments as before, with the exception of $s+1$ instead of s.

The described algorithm terminates automatically as soon as N bond vectors $\mathbf{U}_1$, $\mathbf{U}_2$, ..., $\mathbf{U}_N$ have been created. The coordinates $\{\mathbf{x}\}$ of nodes are simply given by the cumulative sum over the set of bond vectors $\{\mathbf{U}\}$, i.e., $\mathbf{x}_{j+1} = \mathbf{x}_j + \mathbf{U}_j$. Note that using this algorithm the return probabilities satisfy Eq. (1) and that all bond lengths ℓ are automatically confined to the interval $[U_{\rm min}, U_{\rm max}]$ and distributed according to Eq. (2). The proof is provided in the next section.

Algorithm for the generation of a SRRW subject to global cutoff

The idea of a SRRW with global cutoff R_c is to make sure the SRRW will tend to grow within a certain spherical volume of radius $\approx R_c$. To this end the above algorithm

is slightly modified as follows. Instead of the earlier (ii) calculate the geometric center $\mathbf{C}$ of the existing nodes from $\{\mathbf{x}\}$. If $|\mathbf{x}_s - \mathbf{C}| > R_c$, then set $P_R = 1$, otherwise calculate $P_R = |\mathbf{U}_s|^{-\alpha}/\alpha$ as before.

Molecular dynamics protocol for the generation of a SR-EV

A SRRW conformation subject to global cutoff is produced via Monte Carlo as just described; such a conformation usually exhibits a large number of nodes (points) with identical coordinates. All these points need to be turned into beads, i.e., receive a finite spherical volume within the final SR-EV configuration, that should preserve all large scale features and domain characteristics of the SRRW. We alter the local structure to avoid bead-bead overlap, while operating at (ideally) minimal displacement effort. To this end we use the original node coordinates $\{\mathbf{x}\}$ as initial center positions of spherical beads of radius $r_\circ = 0.49$ and unit mass m. In a first step, to allow for a random element, and to avoid center-center distances that are exactly zero up to numerical precision, we displace all overlapping beads randomly by 1% of the bead diameter. Afterwards we employ LAMMPS ***Thompson et al.*** (***2022***) to run a molecular dynamics simulation on the modified SRRW systems composed of spherical beads. We let all beads interact via a soft repulsive radially symmetric pair potential $V(r) = 20\epsilon[1 + \cos(\pi r/r_c)]$ for $r \leq r_c$, and $V(r) = 0$ otherwise, where r denotes the center-center distance between pairs of beads, ϵ the irrelvant energy unit, and the cutoff distance $r_c = 1.03$ is chosen slightly larger than the bead diameter. The system is thermostatted via the Nosé-Hoover scheme at $T = 0.001\,\epsilon/k_B$, and run using a time step $\Delta t = 0.005\,U_{\rm min}\sqrt{m/\epsilon}$. During runtime, the bead-bead pair correlation function $g(r)$ is evaluated at each time step and averaged for a duration of 200 time steps. Each time unit (200 time steps) we inspect the averaged $g(r)$, integrated up to r_c, as this quantity informs about the amount of remaining overlap. In rare cases, the integral did not decrease with time, in that case we start over using another seed value for the random number generator. While the integral keeps decreasing, we monitor the potential energy of the system. As soon as the potential energy has reached a minimum, which happens if the energy is close to zero, we terminate the molecular dynamics run and save the resulting SR-EV coordinates. The minimum center-center distance between pairs of beads in the SR-EV configuration exceeds $2r_\circ$, as we verified. Note that the distribution of bond lengths is significantly different for SR-EV and SRRW conformations.

Proof of the validity of the SRRW algorithm

The forward jump probability $P_J(U) = (\alpha+1)U^{-(\alpha+2)}$ was stated in the manuscript. It was furthermore mentioned that new bonds of length U_1 should not exceed a local dimensionless cutoff length $U_{\rm max}$, while $U_{\rm min} = 1$ within these units. Because P_J is a probability distribution, it must fulfill $\int_1^{U_{\rm max}} P_J(U)dU = 1$ and the properly normalized version thus reads

$$P_J(U) = \frac{(\alpha+1)U^{-(\alpha+2)}}{1 - U_{\rm max}^{-(\alpha+1)}}, \qquad U \in [1, U_{\rm max}]. \tag{3}$$

To efficiently create bond lengths U distributed according to Eq. (3) using equally distributed random numbers $\xi \in [0,1]$, one has to solve the differential equation $\xi'(U) = P_J(U)$ with initial condition $\xi(1) = 0$, and then invert the solution. The solution of the differential equation is $\xi(U) = (U_{\max}/U)^{1+\alpha}(U^{1+\alpha} - 1)/(U_{\max}^{1+\alpha} - 1)$. Solving this expression for U gives $U = (1 - \beta\xi)^{-1/(1+\alpha)}$ with the constant $\beta \equiv 1 - U_{\max}^{-(1+\alpha)}$, so that $U = 1$ and $U = U_{\max}$ for $\xi = 0$ and $\xi = 1$, respectively. This completes the proof of item (A2) with (A1).

It might be just interesting to mention that one has access to some statistical properties of the chain conformation from P_J, while P_R has to be taken into account for the exact calculation. For sufficiently large $U_{\max}$ the mean bond length is

$$\langle U \rangle = \int_1^{U_{\max}} U\, P_J(U)\, dU \;\approx\; \frac{1+\alpha}{\alpha}. \tag{4}$$

For $\alpha \in \{1.1, 1.15, 1.2\}$ the mean bond length is hence $\langle U \rangle \in \{1.91, 1.87, 1.83\}$. Similarly, the mean return probability is approximately

$$\langle P_R \rangle = \int_1^{U_{\max}} P_R(U) P_J(U)\, dU \;\approx\; \frac{1+\alpha}{\alpha(1+2\alpha)}, \tag{5}$$

i.e., $\langle P_R \rangle = \{0.597, 0.567, 0.539\}$ for $\alpha = \{1.1, 1.15, 1.2\}$. While for $\alpha \leq 1.03$ the SRRW basically collapses to a small region in space, beyond this value the effective number of forward steps is approximately $[0.49(\alpha - 1) - 0.02]N \approx (\alpha - 1)N/2$.
